# Soil versus foliar iodine fertilization as a biofortification strategy for field-grown vegetables

**DOI:** 10.3389/fpls.2015.00450

**Published:** 2015-06-23

**Authors:** Patrick G. Lawson, Diemo Daum, Roman Czauderna, Helmut Meuser, Joachim W. Härtling

**Affiliations:** ^1^Department of Plant Nutrition, Faculty of Agricultural Sciences and Landscape Architecture, University of Applied Sciences of OsnabrückOsnabrück, Germany; ^2^Department of Soil Protection and Restoration, Faculty of Agricultural Sciences and Landscape Architecture, University of Applied Sciences of OsnabrückOsnabrück, Germany; ^3^Department of Cultural Studies and Geosciences, Institute of Geography, University of OsnabrückOsnabrück, Germany

**Keywords:** iodine, micronutrient malnutrition, biofortification, soil application, foliar sprays

## Abstract

Iodine (I) biofortification of vegetables by means of soil and foliar applications was investigated in field experiments on a sandy loam soil. Supply of iodine to the soil in trial plots fertilized with potassium iodide (KI) and potassium iodate directly before planting (0, 1.0, 2.5, 7.5, and 15 kg I ha^-1^) increased the iodine concentration in the edible plant parts. The highest iodine accumulation levels were observed in the first growing season: In butterhead lettuce and kohlrabi the desired iodine content [50–100 μg I (100 g FM)^-1^] was obtained or exceeded at a fertilizer rate of 7.5 kg IO_3_^-^-I ha^-1^ without a significant yield reduction or impairment of the marketable quality. In contrast, supplying KI at the same rate resulted in a much lower iodine enrichment and clearly visible growth impairment. Soil applied iodine was phytoavailable only for a short period of time as indicated by a rapid decline of CaCl_2_-extractable iodine in the top soil. Consequently, long-term effects of a one-time iodine soil fertilization could not be observed. A comparison between the soil and the foliar fertilization revealed a better performance of iodine applied aerially to butterhead lettuce, which reached the desired iodine accumulation in edible plant parts at a fertilizer rate of 0.5 kg I^-^-I ha^-1^. In contrast, the iodine content in the tuber of sprayed kohlrabi remained far below the targeted range. The results indicate that a sufficient spreading of iodine applied on the edible plant parts is crucial for the efficiency of the foliar approach and leafy vegetables are the more suitable target crops. The low iodine doses needed as well as the easy and inexpensive application may favor the implementation of foliar sprays as the preferred iodine biofortification strategy in practice.

## Introduction

Iodine plays a vital role in human health and must be regularly supplied in a sufficient quantity to ensure the proper functioning of fundamental physiological processes. The adverse effects of iodine deficiency – intellectual impairment, damaged reproduction, goiter as well as hypo- and hyperthyroidism – are still a serious global public health problem. According to current World Health Organization (WHO) data, almost 1.9 billion individuals worldwide have an inadequate iodine intake. Compared to other WHO regions, Europe has the largest percentage of iodine-deficient people in their population, despite its relative wealth and high standard of health care (Zimmermann and Andersson, 2011; Andersson et al., 2012). Although the use of iodized table salt in households has been well-established in Germany since the 1990s (Scriba et al., 2007), about 40% of school age children still have an insufficient iodine intake (Thamm et al., 2007).

The recommendations by several institutions and organizations for a sufficient daily supply of iodine to adolescents and adults range between 150 and 200 μg I d^-1^; pregnant and lactating women have higher iodine needs amounting to 230 and 260 μg I d^-1^, respectively, (European Food Safety Authority [EFSA], 2006; Institute of Medicine [IOM], 2006; Anke and Arnhold, 2008; Arbeitskreis Jodmangel [AJ], 2013). According to the latest nationwide iodine monitoring, the mean iodine intake of the German population was 119 μg I d^-1^, approximately 30% below the iodine requirements of an adult person (Manz et al., 1998).

The agronomic biofortification of food plants with iodine was suggested as a new strategy to address human iodine deficiency. By applying iodine-containing salts or iodine-rich organic materials (e.g., seaweed) to soils, crops are able to increase the absorption and accumulation of this trace element. Iodine in food is readily bioavailable (up to 99%) and can be easily assimilated (Weng et al., 2014). Tonacchera et al. (2013) investigated the efficiency of iodine prophylaxis in humans by consumption of different biofortified vegetables and recorded a significant increase of the mean urinary iodine excretion, which closely reflects the iodine intake of humans. Furthermore, vegetables biofortified with iodine by means of foliar sprays (Comandini et al., 2013) or soil applications (Weng et al., 2014) showed a high stability of iodine during different domestic cooking procedures. On the contrary, iodine added as iodized table salt to the cooking water of non-biofortified vegetables was subject to substantial losses during the boiling process.

Considering the nutritional iodine gaps in Germany (40–135 μg I d^-1^; Arbeitskreis Jodmangel [AJ], 2009, 2013), a projected iodine concentration range of 50–100 μg I (100 g FM)^-1^ in biofortified vegetables (average portion of 80 g per individual per day) would, to a large extent, cover the dietary iodine deficiency. For higher iodine needs, the vegetable portions could be increased as necessary. The indicated target range seems to be a reasonable concentration level for biofortified vegetables without running the risk of exceeding the upper tolerable intake level (600–1100 μg I d^-1^) for iodine (European Food Safety Authority [EFSA], 2006; Institute of Medicine [IOM], 2006).

Most iodine soil fertilization trials conducted to date were carried out as pot experiments in greenhouses under controlled ambient conditions (Borst Pauwels, 1961; Muramatsu et al., 1989; Dai et al., 2004; Hong et al., 2008). The studies showed characteristic response patterns depending on the oxidation state of the element (I^-^/IO_3_^-^) as well as the applied iodine dose and the examined plant species. Applicable concentrations in the range of 5–25 mg I (kg soil)^-1^ (≈15–75 kg I ha^-1^ in the 0–30 cm soil layer) without yield impairment have been reported (Dai et al., 2004; Hong et al., 2008). Our own preliminary pot trials with butterhead lettuce cultivated in peat growing media showed that concentrations in the range of 1–2.5 mg I (L substrate)^-1^ (≈3–7.5 kg I ha^-1^ in the 0–30 cm soil layer) were sufficient to achieve an appropriate iodine accumulation in edible plant parts. Concentrations of 10 mg I (L substrate)^-1^ (30 kg I ha^-1^ in the 0–30 cm soil layer) induced yield depression.

For the large-scale fertilization of iodine in goiter endemic areas, the iodination of irrigation water was proposed (DeLong et al., 1997; Ren et al., 2008). However, this method requires large amounts of iodine and implies an uncontrolled iodine release into the environment. Another approach could be the incorporation of iodine-containing salts in liquid or granular fertilizers. Commercial products have already been introduced for the fertilization of pastures to improve the iodine supply to grazing livestock (Ravensdown, 2014; Yara, 2014). Practical techniques for the use of straight or compound iodine fertilizers on field vegetables, to enhance the iodine content in edible plant parts to an adequate extent, have not yet been established. Furthermore, little information is available about the duration of the efficacy of a one-time soil fertilization with iodine. Foliar sprays are known to be an efficient alternative to soil fertilization, especially in the case of micronutrients (Eichert and Fernández, 2012). Nevertheless, only a few studies have hitherto been conducted to compare both application methods with regard to iodine biofortification purposes (Smoleñ et al., 2011a,b).

In the present study, trials under open field conditions were conducted, using leafy (butterhead lettuce) and tuber vegetables (kohlrabi/radish), in order to investigate the long-term effects of a one-time iodine soil fertilization and to compare the efficiency of soil versus foliar fertilization techniques.

## Materials and Methods

### Trial Set-Up and Growing Conditions

The experiments were carried out in 2010 and 2011 on sandy loam soil (Sl_3-4_) at the horticultural research station of the University of Applied Sciences, Osnabrück, Germany (site Wulveskamp: N 52° 18′ 41.299^′′^–E 8° 1′ 30.31^′′^). The trials were performed in a split-plot design with three or four replications per treatment and a gross plot area of 4.5–9.5 m^2^. Stock solutions were prepared in the laboratory with pure potassium iodide and potassium iodate (KIO_3_) salts (Ph. Eur. and Rectapur^®^ quality, VWR International GmbH, Bruchsal, Germany). The trial plots were then drenched with diluted stock solutions at different concentrations (0, 1.0, 2.5, 7.5, and 15 kg I ha^-1^), 1 day before planting at a rate of 2 L H_2_O m^-2^. KI and KIO_3_ foliar sprays were prepared from stock solutions and applied once or twice (1 or 1 and 2 weeks) before harvest at different concentrations (1x 0, 1x 0.5, 2x 0.5, 1x 1.0, and 2x 1.0 kg I ha^-1^) at a rate of 600 L H_2_O ha^-1^. The sprayed solutions contained the organosilicone surfactant Break-Thru^®^ S 240 (0.05% v/v; AlzChem AG, Trostberg, Germany) to improve spreading and sticking properties.

Plant material was purchased at Jungpflanzen Lüske GbR, Höltinghausen, Germany. Kohlrabi (*Brassica oleracea* L. var. *gongylodes* L. ‘Lech’) and butterhead lettuce (*Lactuca sativa* L. var. *capitata* cv. ‘Barilla’) seedlings grown in peat substrate were transplanted into soil (2010–2011) within 2 days of delivery. Radish seeds (*Raphanus sativus* L. var. *sativus* cv. ‘Raxe’; Hild Samen GmbH, Marburg, Germany) were sowed in 2011 with a single-seed precision hand-pushed seed drill (Sembdner Maschinenbau GmbH, Fürstenfeldbruck, Germany) at a density of 160 kernel m^-2^.

Entec^®^ 26 (Compo GmbH & Co. KG, Münster, Germany), superphosphate (ICL Fertilizers Deutschland GmbH, Ludwigshafen, Germany) and potassium magnesia (K+S AG, Kassel, Germany) were used to cover the N, P, K, and Mg requirements. The basic N, P, and K fertilization was conducted manually, 3 days before planting or sowing, by spreading the granular fertilizers at the following amounts: 124 kg N ha^-1^, 34 kg P_2_O_5_ ha^-1^, 181 kg K_2_O ha^-1^ (butterhead lettuce in 2010); 204 kg N ha^-1^, 46 kg P_2_O_5_ ha^-1^, 190 kg K_2_O ha^-1^ (kohlrabi in 2010); 110 kg N ha^-1^, 21 kg P_2_O_5_ ha^-1^, 101 kg K_2_O ha^-1^ (radish in 2011); 150 kg N ha^-1^, 34 kg P_2_O_5_ ha^-1^, 181 kg K_2_O ha^-1^ (butterhead lettuce in 2011).

Climatic data were collected at the horticultural research station (for detailed data refer to Lawson, 2014). The Osnabrück region, located in south-western Lower-Saxony, is generally characterized by a warm-moderate climate with mild winters and cool summers. The long-term averages for minimum and maximum air temperature, rainfall and rain days are 1.8°C (January), 17.6°C (July), 865 mm, and 122 days, respectively, (Deutscher Wetterdienst [DWD], 2013).

### Tissue Total Iodine Determination

The plant material harvested for iodine determination was washed thoroughly with tap water in order to imitate a common domestic cleaning process: each sample was immerged in water and excess water removed with a salad spinner, then again thoroughly flushed with tap water and excess water removed. The samples were then transferred to a desiccating oven with air recirculation and dried at 60°C until weight constancy. The dried plant material was finely ground using a 500 μm sieve in an ultra-centrifugal rotor mill (model ZM 100, Retsch GmbH, Haan, Germany). Just before chemical digestion, the samples were dried again overnight at 60°C in a desiccating cabinet and re-cooled to room temperature.

The alkaline digestion method used was adapted from the procedures described by Jopke et al. (1997) and Kučera and Krausová (2007). Briefly, 0.100 g of the plant material were weighed in Sigradur glassy carbon crucibles (type GAT 4, HTW GmbH, Thierhaupten, Germany) and 1.678 mL of the KOH solution (Emsure^®^, 47% v/v, Merck KGaA, Darmstadt, Germany) were added. The crucibles were covered with a watch glass and then subjected to a stepwise heating procedure: up to a maximum of 300°C was achieved on a Trio-Term precision aluminum hot plate and then up to 450°C on a Ceran hot plate (model C 450, C. Gerhardt GmbH & CO. KG, Königswinter, Germany). Subsequently, the crucibles were placed in a muﬄe furnace at 550°C and, after cooling, the fusion cake was solubilized by adding deionized water and placing the crucibles in an ultrasonic bath (model Sonorex^®^ RK 255 H, Bandelin electronic GmbH & Co. KG, Berlin, Germany). The solution was then quantitatively transferred to volumetric flasks (100 mL) by rinsing the crucibles and the watch glasses with deionized water (resulting in a 0.2 M KOH matrix).

Iodine detection was performed according to the Quick-Chem method 10-136-09-1-A (Switala, 2001), using a flow injection analysis (FIA) system model Quick-Chem^®^ 8500 equipped with an automated diluter and sampler, an iodide manifold and the Omnion^®^ 2.2.2 software (all components from Lachat Instruments, Hach Company, Loveland, CO, USA). The quality control of the analytical data was established by running a method comparison (Quick-Chem method 10-136-09-1-A compared to the DIN EN 15111 method; Thüringer Umweltinstitut, Henterich GmbH & Co. KG, Krauthausen, Germany) and a subsequent correlation analysis. A recovery rate of 92.5%, which is comparable to the findings of Johner et al. (2012), was found in samples within an iodine concentration range of 2–190 μg I L^-1^.

### Determination of Calcium Chloride Extractable Iodine in Soil

The calcium chloride extractable iodine fraction in soil samples was extracted at a ratio of 1 + 4 (m + v) adapting the extraction method suggested by Altinok et al. (2003), which is analogous to the procedure of the N_min_ method to determine the mineral nitrogen content of soils (VDLUFA, 1997). 150.00 g of soil (at actual field moisture levels) were suspended in 600 mL of a 0.0125 *M* CaCl_2_-solution in 1000 mL PE-bottles and stirred mechanically for 1 h on a reciprocal motion shaker (model Laboshake^®^ LS 500, C. Gerhardt GmbH & CO. KG, Königswinter, Germany). The soil sample duplicates were then filtrated through a folded filter (type MN 619 G ¼, Macherey-Nagel GmbH & Co. KG, Dueren, Germany) and the first 100 mL of the percolate were discarded. Due to an incompatible matrix, the iodine contents in calcium chloride extracts could not be detected by FIA and were, therefore, determined by using inductively coupled plasma mass spectrometry (ICP-MS) according to the DIN EN ISO 17294-2 method. This analysis was carried out by an external laboratory (Thüringer Umweltinstitut, Henterich GmbH & Co. KG, Krauthausen, Germany).

### Statistical Procedures

Iodine concentration data were subjected to a one-way ANOVA and, if needed, to a normalization procedure by either using logarithmic, square root or Box–Cox transformations. If normalization was not possible, the data was transformed into ranks and then analyzed by means of parameter-free methods (Wilcoxon rank-sum test and Friedman’s rank test if balanced data was available).

Additionally, a multifactorial GLM ANOVA was performed to test the trial factors and their interaction (iodine form, iodine dose, form × dose). The Bonferroni multiple comparison procedure at α = 0.05 was used to compare the means. All the statistical tests were conducted, with the exception of the Box–Cox transformation (SPSS^®^ 20), using the program NCSS 2007.

## Results

### Yield and Marketable Quality

**FIGURE 1:**
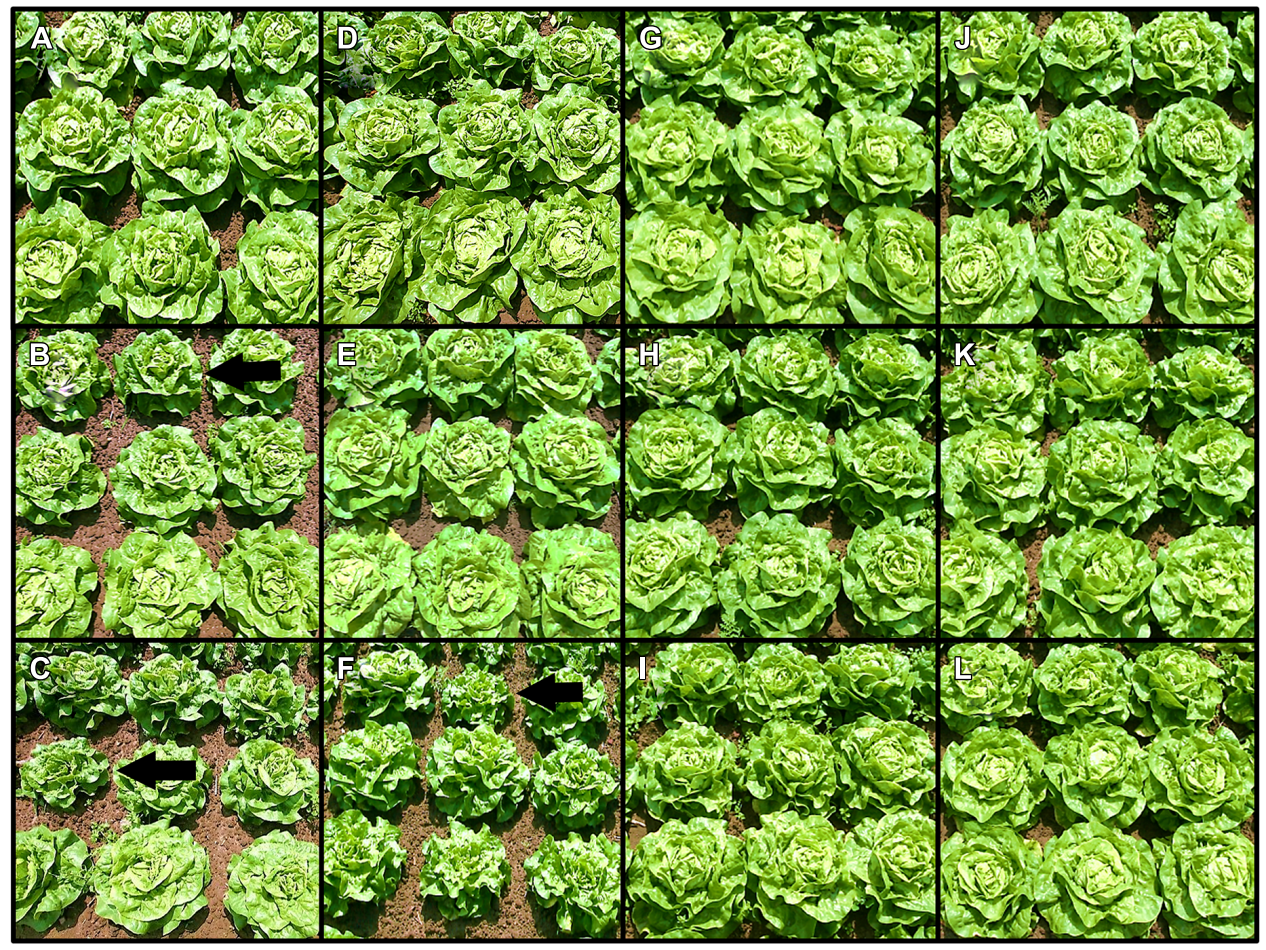
**A visual comparison of butterhead lettuce 1 day before harvest, cultivated in 2010 using different application methods, iodine doses and forms.** Soil application in season 2010: **(A)** 0 kg I^-^-I ha^-1^, **(B)** 7.5 kg I^-^-I ha^-1^, **(C)** 15 kg I^-^-I ha^-1^, **(D)** 0 kg IO_3_^-^-I ha^-1^, **(E)** 7.5 kg IO_3_^-^-I ha^-1^, **(F)** 15 kg IO_3_^-^-I ha^-1^. Foliar application in season 2010: **(G)** 0 kg I^-^-I ha^-1^, **(H)** 1 kg I^-^-I ha^-1^, **(I)** 2x 1 kg I^-^-I ha^-1^, **(J)** 0 kg IO_3_^-^-I ha^-1^, **(K)** 1 kg IO_3_^-^-I ha^-1^, **(L)** 2x 1 kg IO_**3**_^-^-I ha^-1^. The black arrows indicate smaller heads within a single plot.

**FIGURE 2:**
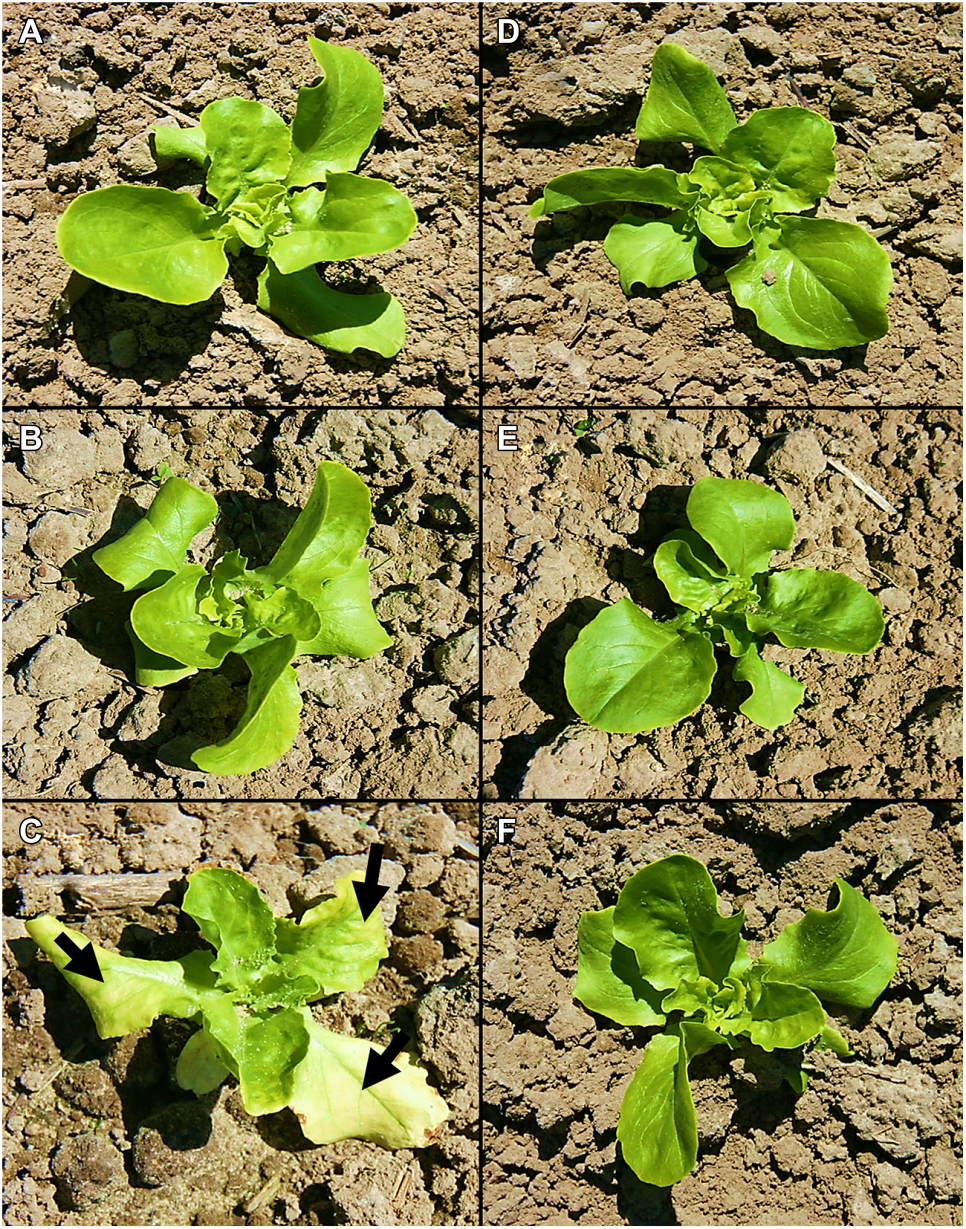
**A visual comparison of butterhead lettuce soil cube transplants 8 days after planting at different iodine doses applied by means of soil drenches. (A)** 0 kg I^-^-I ha^-1^, **(C)** 7.5 kg I^-^-I ha^-1^, **(E)** 15 kg I^-^-I ha^-1^, **(B)** 0 kg IO_3_^-^-I ha^-1^, **(D)** 7.5 kg IO_3_^-^-I ha^-1^, **(F)** 15 kg IO_3_^-^-I ha^-1^. The black arrows indicate chlorotic intercostal areas or necrotic spots.

The influence of different iodine fertilization techniques on crop yield and marketable quality was investigated using butterhead lettuce and kohlrabi/radish as model crops. After a one-time soil fertilization of KI and KIO_3_, differences in biomass production were not statistically significant in any case (**Table 1**). However, in the first growing season, the lowest mean crop yields were noticed at the highest iodine supply level. At the same time, the crop population became more inhomogeneous compared to the control plants. **Figures 1A–F** illustrate for butterhead lettuce the average development of head size and variability as affected by increasing fertilizer doses. A few days after planting, butterhead lettuce transplants cultivated at 15 kg I^-^-I ha^-1^ developed chlorotic leaves with yellow intercostal leaf areas (**Figure 2C**) turning increasingly into necrotic spots. Although showing growth inhibition, most plantlets recovered within a short period of time. An iodine supply up to 7.5 kg I ha^-1^ applied as KIO_3_ did not affect growth or the marketable quality of the investigated crops.

**Table 1:** Influence of a one-time iodine fertilization over two seasons, applied in 2010 by means of soil drenches, on the yield of selected crops.

Crop	Kohlrabi		Butterhead lettuce				Radish	
Cultivation year	2010				2011			
Iodine form	I^-^	IO_3_^-^	I^-^	IO_3_^-^	I^-^	IO_3_^-^	I^-^	IO_3_^-^
**Treatment dose [kg I ha^-1^]**	**Relative crop yield [%]**							
0	100.0 a	100.0 a	100.0 a	100.0 a	100.0 a	100.0 a	100.0 a	100.0 a
1.0	90.2 a	98.6 a	97.9 a	92.0 a	103.1 a	93.9 a	108.6 a	94.0 a
2.5	93.1 a	106.5 a	91.7 a	94.3 a	99.4 a	105.5 a	94.4 a	100.0 a
7.5	79.6 a	100.2 a	85.2 a	94.2 a	113.4 a	102.1 a	85.8 a	97.1 a
15	72.1 a	95.5 a	73.6 a	72.7 a	107.0 a	98.1 a	90.5 a	89.2 a

One-way ANOVA (*p*-value)	NS (0.181)		NS (0.389)		NS (0.992)		NS (0.059)	

Fresh matter yield is expressed as percent of the unfortified control treatment (*n* = 3). Percentages with same letters do not differ (contrast calculated between sources) according to Bonferroni MCP at *a* = 0.05. Levels of significance are represented by ^∗^*p* < 0.05, ^∗∗^*p* < 0.01, and ^∗∗∗^*p* < 0.001, and NS, not significant = *p* > 0.05.

In the case of foliar sprays (KI and KIO_3_ applied once or twice at a rate of 0.5 or 1.0 kg I ha^-1^), the biomass production and the marketable quality of butterhead lettuce and kohlrabi were not affected significantly by the iodine supply (**Table 2**). A visual comparison of the foliar sprays applied to butterhead lettuce also indicated no noticeable differences from the unfortified control in the overall head size with increasing fertilizer dose (**Figures 1G–L**).

**Table 2:** Comparison of iodine application methods (soil drenches and foliar sprays applied 2010) on the crop yield of kohlrabi and butterhead lettuce.

Crop			Kohlrabi				Butterhead lettuce			
Application method			Soil drenches		Foliar sprays		Soil drenches		Foliar sprays	
Iodine form			I^-^	IO_3_^-^	I^-^	IO_3_^-^	I^-^	IO_3_^-^	I^-^	IO_3_^-^
**Treatment dose [kg I ha^-1^]**							
**Soil drenches**	**Foliar sprays**		**Relative crop yield [%]**							
0	0		100.0 a	100.0 a	100.0 a	100.0 a	100.0 a	100.0 a	100.0 a	100.0 a
1.0	0.5		90.2 a	98.6 a	100.6 a	93.5 a	97.9 a	92.0 a	98.3 a	107.4 a
2.5	2x 0.5		93.1 a	106.5 a	104.7 a	98.6 a	91.7 a	94.3 a	89.5 a	99.2 a
7.5	1.0		79.6 a	100.2 a	99.7 a	97.4 a	85.2 a	94.2 a	94.9 a	99.2 a
15	2x 1.0		72.1 a	95.5 a	98.0 a	95.8 a	73.6 a	72.7 a	86.8 a	91.9 a

One-way ANOVA (*p*-value)			NS (0.181)		NS (0.875)		NS (0.389)		NS (0.099)	

Fresh matter yield is expressed as percent of the unfortified control treatment. Soil drenches: *n* = 3; foliar sprays: *n* = 4. Percentages with same letters do not differ (contrast calculated between sources) according to Bonferroni MCP at *a* = 0.05. Levels of significance are represented by ^∗^*p* < 0.05, ^∗∗^*p* < 0.01, and ^∗∗∗^*p* < 0.001 and NS, not significant = *p* > 0.05 (actual probability level).

### Influence of a One-Time Iodine Soil Fertilization Over Two Growing Seasons

**FIGURE 3:**
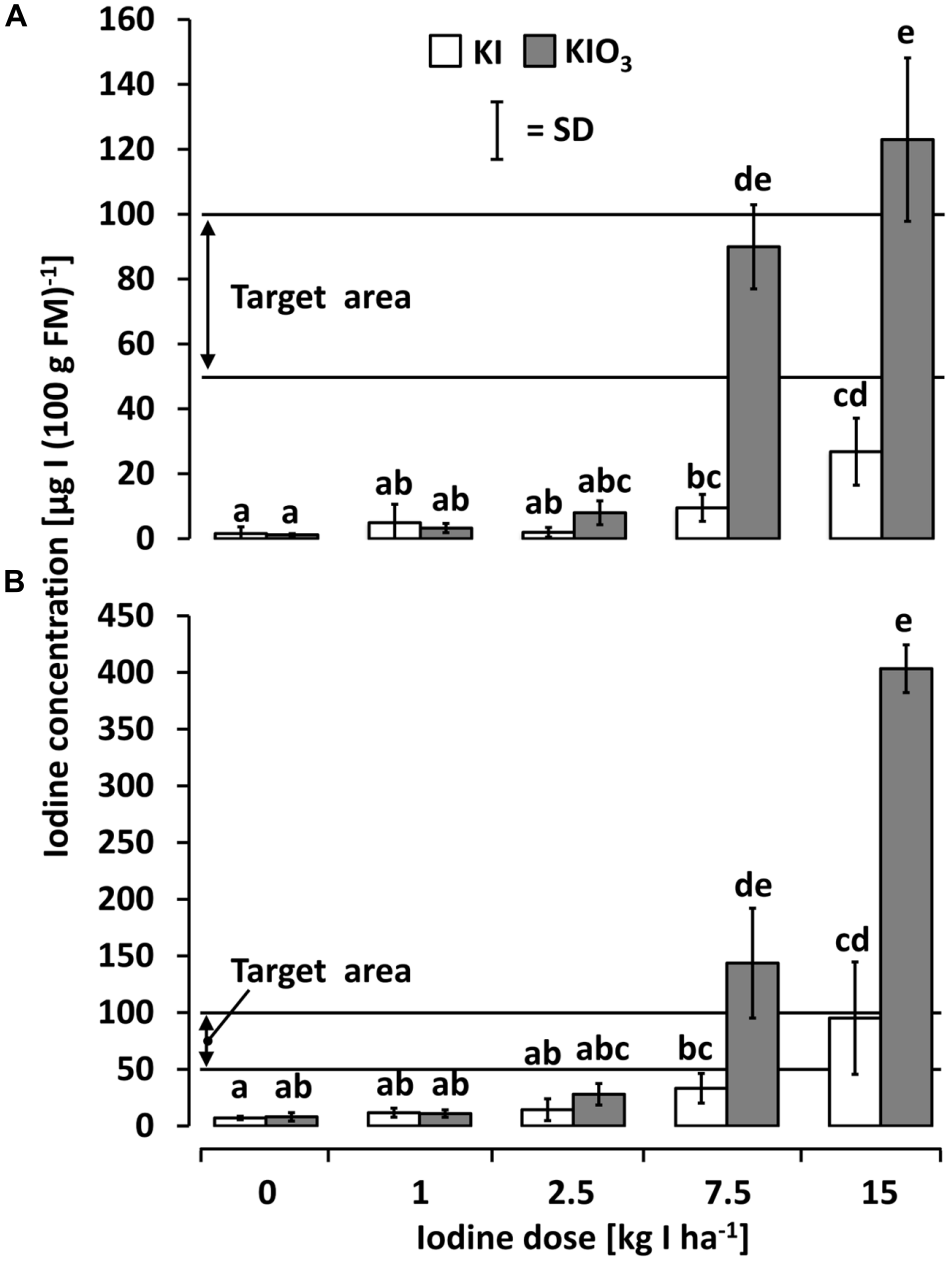
**The iodine accumulation behavior of **(A)** kohlrabi and **(B)** butterhead lettuce cultivated in season 2010 after a one-time iodine soil fertilization applied 2010.** Means with same letters do not differ according to Bonferroni MCP at *a* = 0.05 [One-way analysis of variance **(A)**: probability level = 0.00, power = 1.00; One-way analysis of variance **(B)**: probability level = 0.00, power = 1.00]. *n* = 3.

**Figure 3A** shows the iodine accumulation behavior of kohlrabi cultivated in the first season after a single iodine soil fertilization applied just before planting in 2010. An increasing iodine content in edible plant parts was observed with a rising iodine supply, particularly when using KIO_3_ as the iodine fertilizer. The desirable iodine amount in edible plant parts was achieved at ≥7.5 kg IO_3_^-^-I ha^-1^. KI treatments were less effective and did not reach the target range [50–100 μg I (100 g FM)^-1^] in any variant using kohlrabi as a model crop.

A similar pattern, but at higher accumulation levels, was found in butterhead lettuce cultivated in 2010 (**Figure 3B**). Again, an increasing iodine content with rising iodine supply and a higher accumulation tendency of trial variants treated with KIO_3_ was observed. Significant differences to the unfortified control occurred at ≥7.5 kg IO_3_^-^-I ha^-1^ where the intended iodine level was exceeded to the extent of 50–300%. In KI-treatments at the same iodine doses, a significantly lower iodine accumulation was found; the target range was reached at only 15 kg I^-^-I ha^-1^.

**FIGURE 4:**
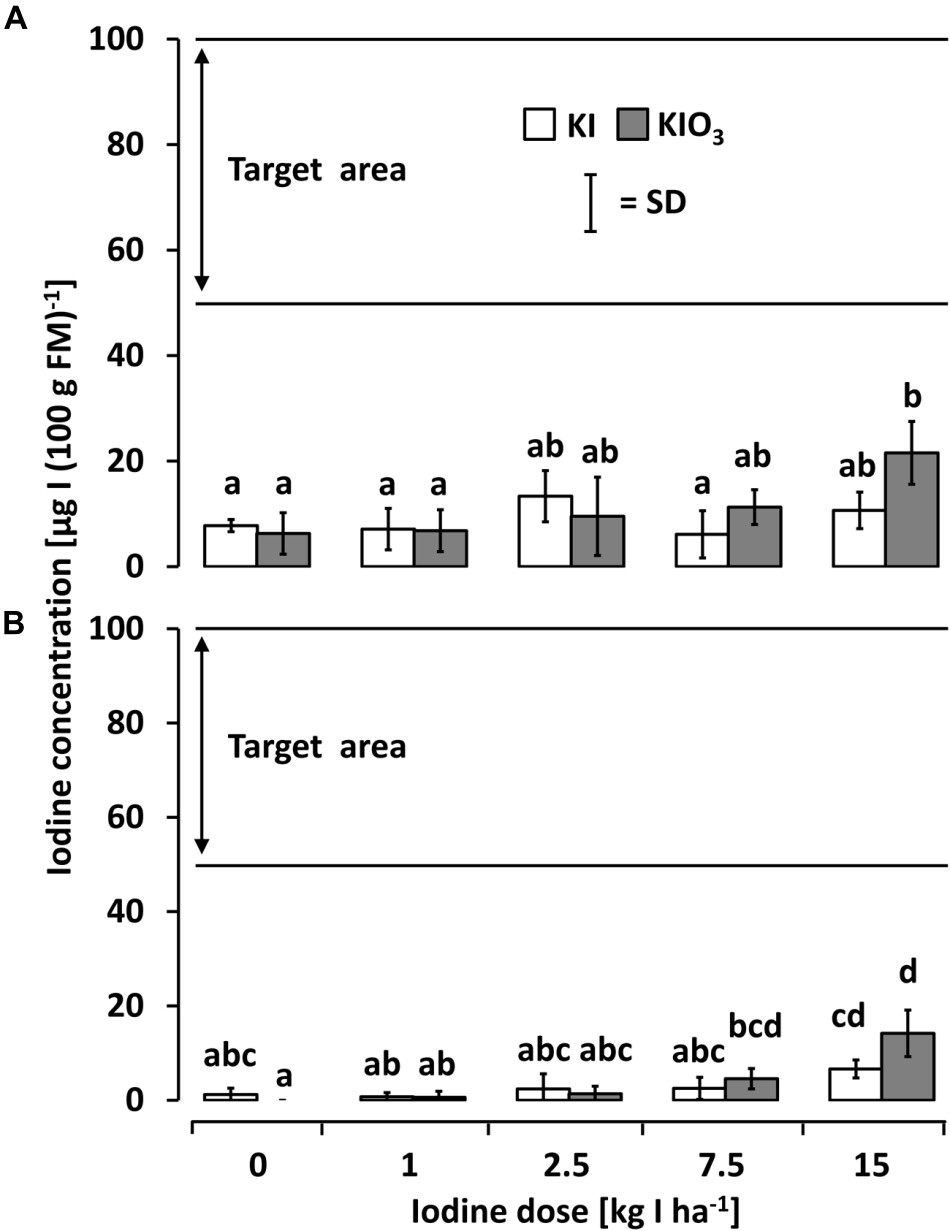
**The iodine accumulation behavior of **(A)** butterhead lettuce and **(B)** radish cultivated in season 2011 after a one-time iodine soil fertilization applied 2010.** Means with same letters do not differ according to Bonferroni MCP at *a* = 0.05 [One-way analysis of variance **(A)**: probability level = 0.037115, power = 0.805387; one-way analysis of variance **(B)**: probability level = 0.000012, power = 0.99999]. *n* = 3.

In the second season (2011), the rotational crops cultivated on the same plots (butterhead lettuce after kohlrabi and radish after butterhead lettuce) without further iodine fertilization showed only little or no iodine accumulation (**Figures 4A,B**). In both succeeding crops, a single significant difference to both unfortified controls was found in plots fertilized with 15 kg IO_3_^-^-I ha^-1^ one year before. Even at this dose, the recorded iodine accumulation was distinctly below the desired range.

**FIGURE 5:**
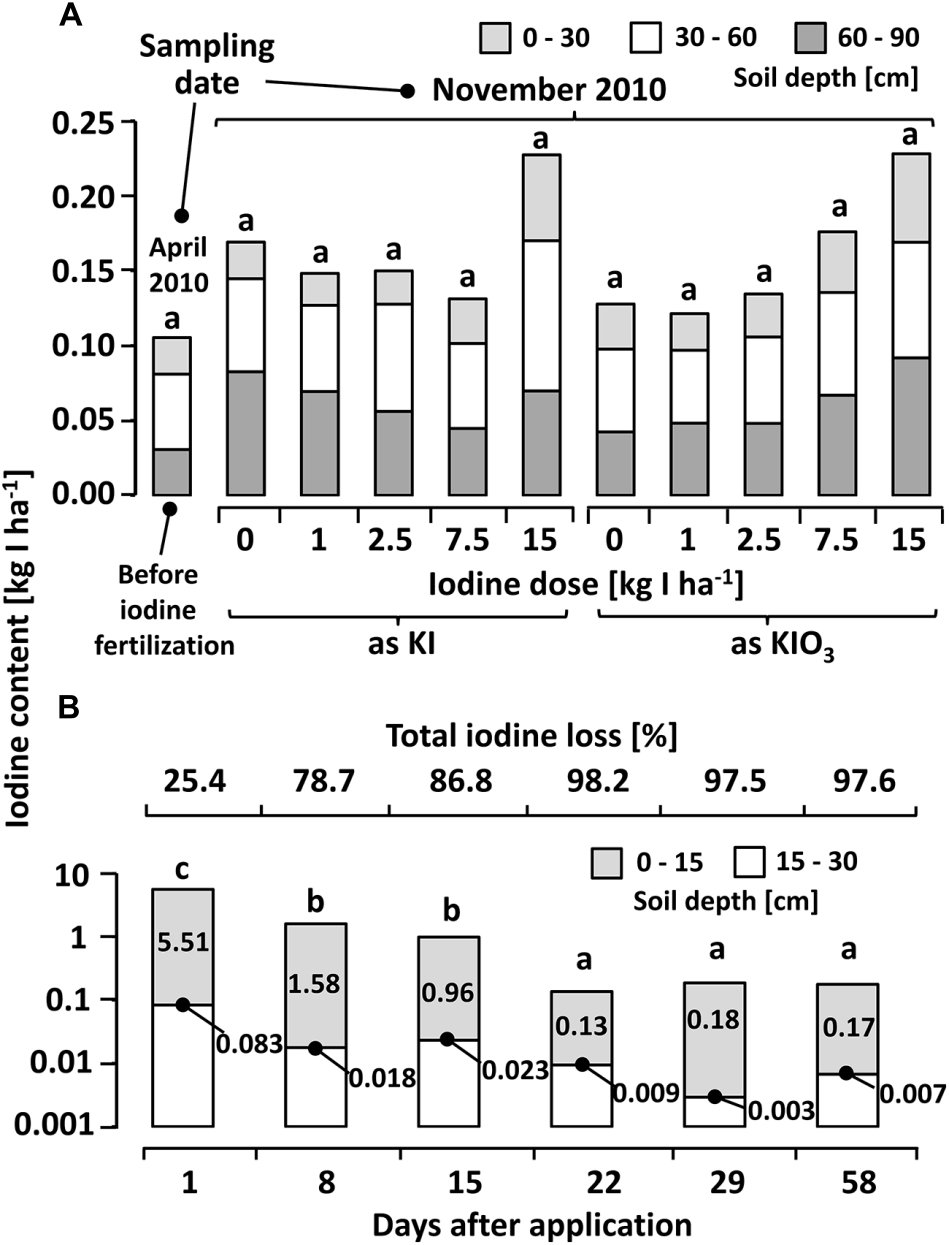
**(A)** Calcium chloride extractable iodine (12.5 mM CaCl_2_ solution) at different depths in a sandy loam soil (Sl_3_) before and six months after iodine fertilization by means of soil drenches at different concentrations. Means with same letters do not differ according to Bonferroni MCP at α = 0.05 (One-way analysis of variance: probability level = 0.053, power = 0.78). *n* = 3. **(B)** Calcium chloride extractable iodine in soil samples collected at different intervals after a one-time iodine fertilization at 7.5 kg IO_3_^-^-I ha^-1^ (One-way analysis of variance: probability level = 0.00, power = 1.00). *n* = 4.

Only little differences were recorded in the CaCl_2_-extractable iodine content of soil samples collected before and 6 months after the iodine soil fertilization (**Figure 5A**). Although a slight increase of iodine concentration in the soil was observed with increasing iodine fertilizer doses (especially soil depths of 60–90 cm in case of KIO_3_), no statistically significant differences to the control treatment or to the ambient level value before the fertilization occurred, were detected.

The decrease in iodine recovery following a one-time iodine fertilization with 7.5 kg IO_3_^-^-I ha^-1^ at shorter sample collection intervals was remarkable (**Figure 5B**). A rapid reduction in the CaCl_2_-extractable iodine concentration was recorded within the first week. Three weeks after the initial application, the majority of the exogenously applied iodine was no longer detectable in the top soil without indications of iodine displacement in the deeper soil layer (15–30 cm).

### Comparison between the Soil and Foliar Application Method

**FIGURE 6:**
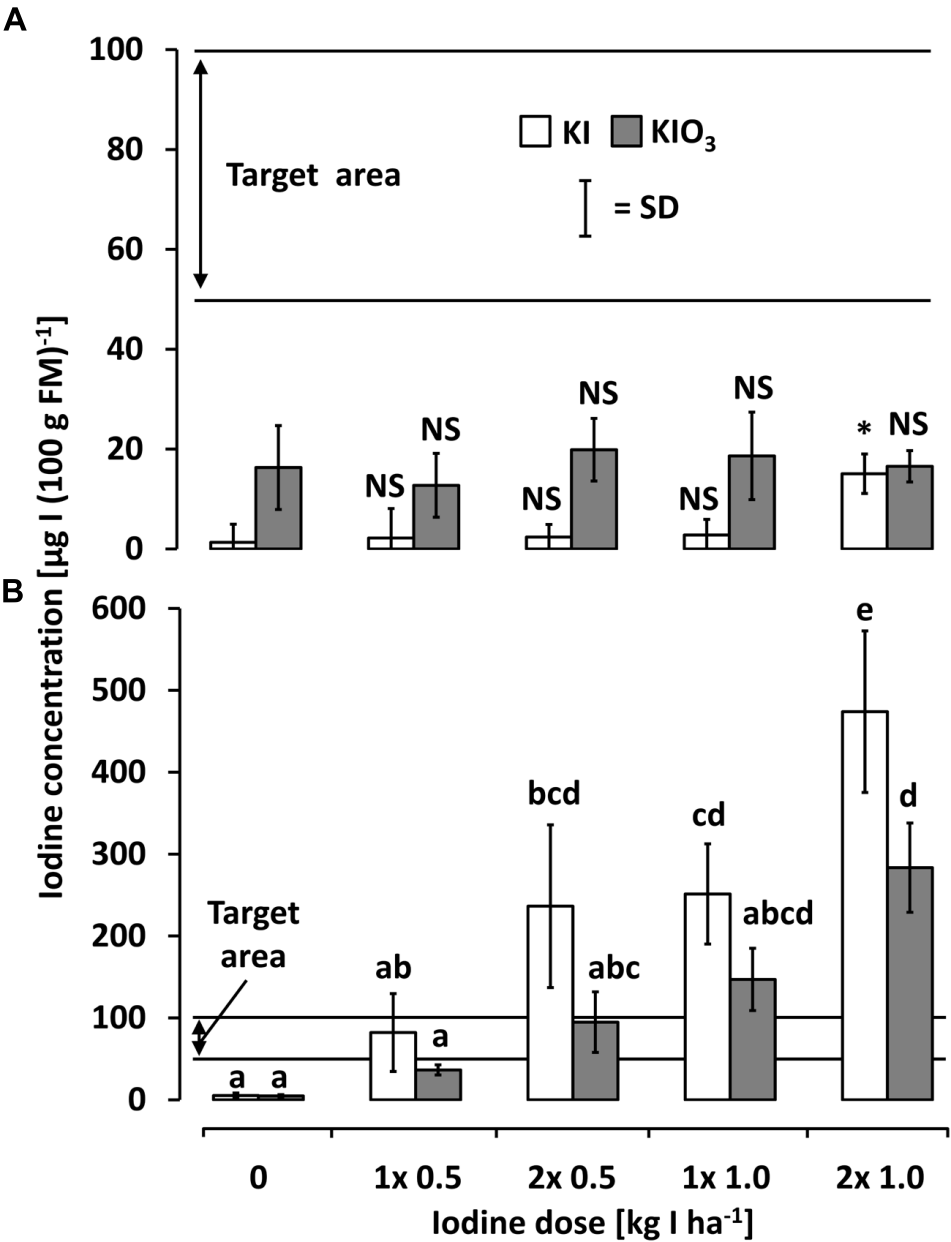
**The iodine accumulation behavior of **(A)** kohlrabi and **(B)** butterhead lettuce as affected by iodine foliar sprays at varied concentrations.** Means with same letters do not differ according to Bonferroni MCP at *a* = 0.05 Levels of significance are represented by ^∗^*p* < 0.05, ^∗∗^*p* < 0.01, ^∗∗∗^*p* < 0.001, and NS, not significant = *p* > 0.05. Kohlrabi foliar spray data (non-normal) was transformed into ranks and compared to respective control by Wilcoxon rank-sum test. *n* = 4.

**Figure 6** shows the iodine accumulation behavior of kohlrabi and butterhead lettuce as affected by iodine foliar sprays. Generally, the foliar sprays applied to kohlrabi did not lead to a substantial iodine accumulation in edible plant parts. In all cases, this was far below the desired minimal amount of 50 μg I (100 g FM)^-1^. The non-parametric comparison to the control level showed a slight iodine enhancement when applying KI at the highest dose.

Foliar spray treatments on butterhead lettuce led to very high iodine accumulation levels exceeding the soil application results. In contrast to the soil drenches, a more pronounced iodine accumulation tendency was observed in the iodide treatments (**Figure 6B**). The targeted accumulation range could already be reached at the lowest doses of 0.5 kg I^-^-I ha^-1^ and 2x 0.5 kg IO_3_^-^I ha^-1^.

## Discussion

### Yield and Marketable Quality

No statistically significant differences in biomass production could be attributed to a one-time KI or KIO_3_ soil application. However, after a fertilization of 15 kg I ha^-1^, the mean crop yield of butterhead lettuce and kohlrabi was up to 28% lower compared to the control plants (**Table 1**). Furthermore, at this iodine dose the head size of butterhead lettuce was visibly reduced and the crop population became more inhomogeneous (**Figure 1**). Phytotoxic symptoms, including chlorosis and necrotic spots on older leaves, were observed in the very early developmental stage of butterhead lettuce transplants when the highest KI dose was applied (**Figure 2**). Considering that soil drenches presumably infiltrated only a few centimeters (1.5–3 cm) into the soil directly after the iodine fertilization (Tanaka et al., 2012), the initial iodine concentration was in the range of approximately 33–66 mg I^-^-I (kg soil)^-1^ and could have caused the depicted detrimental effects. This would be in concordance with the observations of Hong et al. (2008) and Weng et al. (2008) on different vegetable species and the stated deleterious effects of an iodine dose of ≥50 mg I^-^-I (kg soil)^-1^.

Little is known about the mechanism of iodide toxicity in plants. However, at least in part, it may arise from intracellular oxidation of iodide to elemental iodine followed by iodination of cellular components, including chlorophyll (Mynett and Wain, 1973). Soil applied KIO_3_ did not induce phytotoxic symptoms in butterhead lettuce transplants (**Figure 2**). Furthermore, mean crop yield was hardly affected by iodate supply, especially in the case of kohlrabi (**Table 1**). These observations are in agreement with previous reports, indicating that the influence of iodide on plant growth is more adverse than iodate (Mackowiak and Grossl, 1999; Zhu et al., 2003; Blasco et al., 2008; Caffagni et al., 2011).

The inhomogeneous plant growth within the single plots (especially at the highest iodine treatments) and noticeable fluctuations in crop yield between the blocks (due to the sloped and heterogeneous arable field at the trial site) affected data variation. Consequently, the noticed reduction in biomass production was not statistically significant in any case (**Table 1**). Despite of this variation in crop yield, the results of the soil fertilization experiments clearly indicate that a moderate iodine dose of up to 7.5 kg I ha^-1^ applied as KIO_3_ is well tolerated by butterhead lettuce and kohlrabi. On the other hand, a stimulation of plant growth following low iodine application rates, as occasionally reported in the literature (Hong et al., 2008; Weng et al., 2008), could not be observed in our trials.

The visual assessment of butterhead lettuce (**Figure 1**) treated with foliar sprays showed, in comparison to the soil iodine application, a much more homogenous head size across all treatments. No adverse effects were noticed on leaves and mean crop yield was unaffected up to a total application amount of 1 kg I ha^-1^ (**Table 2**). In accordance with these observations, Smoleñ et al. (2011a) also found no significant differences in biomass production in similar field trials on butterhead lettuce. Although statistically not significant, Altinok et al. (2003) reported yield promotion of alfalfa forage using potassium iodide foliar sprays (1–2 kg I^-^-I ha^-1^). In contrast, Strzetelsky et al. (2010) stated yield depression after spraying radish with KI at 2 × 0.8 kg I ha^-1^. Apparently, the growth response to aerially applied iodine is different between plant species, but total doses of >1 kg I ha^-1^ may be critical.

### Efficiency of a One-Time Iodine Soil Fertilization Over Time

The native iodine concentration of the fresh vegetables investigated in this study ranged between 1.2 and 16.3 μg I (100 g FM)^-1^ and thus was in accordance with values reported previously in literature (Anke et al., 1993; Fordyce, 2003; Haldimann et al., 2005). After a one-time iodine soil fertilization at rates of ≥7.5 kg IO_3_^-^-I ha^-1^ butterhead lettuce and kohlrabi accumulated iodine in their edible plant parts to a satisfactory amount [≥50 μg I (100 g FM)^-1^]. In contrast, vegetables grown on the same plots in the second season without further iodine treatment did not accumulate iodine to an adequate extent (**Figure 4**). Thus, long term effects of iodine application by means of soil drenches could not be observed.

The response of vegetables to a one-time iodine soil fertilization was conditioned primarily by the applied iodine form and dose, the crop used and the elapsed time. Higher iodine concentrations in vegetables were found throughout in the KIO_3_ treatments in the experiments. This result is consistent with the findings of Dai et al. (2006) and Weng et al. (2008) who also reported distinctively higher iodine accumulation fertilizing with the oxidized iodine form. The inherent difference between the two iodine forms is likely to be a result of the higher mobility and turnover of I^-^ in soils. Iodide leaches from the root zone more quickly, is readily fixed in humus and rapidly volatilizes in the form of organoiodides such as methyliodide (Muramatsu and Yoshida, 1995; Fuge, 1996; Redeker et al., 2000; Dimmer et al., 2001; Johnson, 2003). Consequently, iodide is either more rapidly lost after its application to soils or remains less phytoavailable in the root zone than iodate. On the other hand, studies on plants grown in hydroponic systems have shown that roots absorb I^-^ at a higher rate than IO_3_^-^ (Zhu et al., 2003; Blasco et al., 2008; Voogt et al., 2010). This is attributed to the heavier molecular weight and the higher valence of iodate (Mackowiak and Grossl, 1999). Furthermore, it is supposed that IO_3_^-^ is reduced to I^-^ before its uptake by plant roots and thus the slow absorbtion rate of iodate is limited by the reduction process (Böszörmènyi and Cseh, 1960; Zhu et al., 2003). Recent investigations on rice indicate that the iodate reduction activity in roots responds to the external iodine concentration (Kato et al., 2013). Overall, it seems that I^-^ is more readily plant available in the solution of soilless culture systems whereas, under field conditions, it is more subject to cumulative losses than IO_3_^-^.

Butterhead lettuce and kohlrabi showed a remarkable difference in the iodine accumulation behavior: Butterhead lettuce proved to be a very good iodine accumulator since this crop distinctly exceeded the desired iodine concentration in edible plant parts at the highest fertilization rate. Hence, an amount of approximately 5 kg IO_3_^-^-I ha^-1^ would probably be sufficient to reach the target level range. Although kohlrabi had a less accentuated response to the iodine treatments, satisfactory results were achieved at 7.5 kg IO_3_^-^-I ha^-1^.

The soil analyses after the first cultivation season (**Figure 5A**) revealed a large decrease in CaCl_2_-extractable iodine. This explains the low iodine accumulation in crops in the second season and emphasizes the short-term availability of the fertilized iodine in soil. The postponed trial with a soil sample collection at short intervals (**Figure 5B**) showed that, even when fertilizing iodine in its oxidized form, a very quick and significant iodine loss without displacement into the deeper soil layers occurs. Hence, the leaching of iodine seems to be, at least for the tested soil (Sl_3-4_), a minor pathway of loss, which confirms similar observations by Weng et al. (2009). Other potential iodine sinks may be, as mentioned above, the microbial formation of organoiodides, the fixation of iodine into the soil organic matter as well as its adsorption on iron and aluminum oxides (Whitehead, 1978, 1981, 1984). In addition, the oxidizing effect of different bacterial strains and enzymes may be of importance for the immobilization of iodine in soils (Muramatsu and Amachi, 2007; Shimamoto et al., 2011; Suzuki et al., 2012).

Regardless of the reasons for the observed iodine dynamic in the soil, the changes occurred very quickly. Within three weeks, the concentration of CaC_2_-extractable iodine declined to the ambient level of the soil (**Figure 5B**). This development affected the crops investigated during the first growing season to a different extent: The faster developing butterhead lettuce spent a higher percentage of its total cultivation period (6 weeks) in an iodine-enriched substrate compared to kohlrabi (total cultivation period 8 weeks). Hence, the iodine application point in time in relation to the growth pattern of the vegetable species has to be recognized as a crucial factor, since long-term effects through iodine fertilization cannot be expected.

### Efficiency Comparison Between the Soil and the Foliar Application Method

The different application techniques – soil vs. foliar fertilization – revealed a pronounced difference in the iodine accumulation behavior depending on the vegetable species studied. Butterhead lettuce showed higher accumulation rates when applying iodine by means of foliar sprays. In contrast to soil application (**Figure 3B**), a higher iodine accumulation in the edible parts was observed using KI as the iodine fertilizer (**Figure 6B**) and the targeted iodine content was already obtained at the lowest fertilizer rate of 0.5 kg I^-^-I ha^-1^. Thus, the foliar fertilization technique was distinctly more efficient for the iodine biofortification of butterhead lettuce and it can be assumed that a number of other leafy vegetables will show a similar response, as investigations on a selection of vegetables crops have shown (Lawson, 2014). However, Smoleñ et al. (2011a) achieved a statistically significant difference in iodine accumulation spraying butterhead lettuce only at the highest fertilization dose of 4x 2 kg IO_3_^-^-I ha^-1^. This deviating result may be explained by a rather high iodine level in the control treatment (about 10 times higher than in our study) and a lack of surfactants in the foliar spray solutions.

The post-harvest treatment chosen in our investigations included thorough washing of the produce under flowing tap water and thus was in accordance with the domestic cleaning process commonly used for fresh vegetables. Therefore, the results for butterhead lettuce are reflecting the actual iodine concentration in ready-to-eat salads. The share of iodine which is only weakly adhering on the leaf surface will presumably be removed by the described procedure. However, investigations with the aid of radioiodine showed that foliar applied I could be washed off only within a few hours after application (Oestling et al., 1989), thus indicating that aerially applied iodine may be rapidly absorbed by leaves. Furthermore, greenhouse trials with potted herbs sprayed with KI and KIO_3_ showed no significant differences between thoroughly washed and unwashed samples (Lawson, 2014).

In the case of kohlrabi, satisfactory results in iodine accumulation could only be achieved by means of soil fertilization (**Figure 3A**). The low iodine levels found in the kohlrabi stem tuber as a result of foliar sprays (**Figure 6A**) indicate a marginal iodine phloem mobility as previously reported by other authors (Herrett et al., 1962; Blasco et al., 2008; Voogt et al., 2010). Furthermore, the highly hydrophobic leaf cuticle layers of kohlrabi may have substantially constrained the uptake of iodine and, consequently, limited its translocation into the edible plant part.

## Conclusion

Our results demonstrate that iodine foliar sprays are a suitable method to increase the iodine content of butterhead lettuce to an appropriate level without yield reduction or impairment in the marketable quality. The low iodine doses needed as well as the easy and inexpensive application of foliar sprays may favor its implementation in practice. Therefore, this iodine biofortification approach should be further elaborated, especially for leafy vegetables.

## Conflict of Interest Statement

The authors declare that the research was conducted in the absence of any commercial or financial relationships that could be construed as a potential conflict of interest.
